# How many hospitalizations has the COVID-19 vaccination already prevented in São Paulo?

**DOI:** 10.6061/clinics/2021/e3250

**Published:** 2021-08-27

**Authors:** Rafael Izbicki, Leonardo S. Bastos, Meyer Izbicki, Hedibert F. Lopes, Tiago Mendonça dos Santos

**Affiliations:** I Departamento de Estatistica, Universidade Federal de Sao Carlos, Sao Carlos, SP, BR.; II Programa de Computacao Cientifica, Fundacao Oswaldo Cruz, Rio de Janeiro, RJ, BR.; III Laboratorio de Funcao Pulmonar, Divisao Respiratoria, Departamento de Medicina, Escola Paulista de Medicina, Universidade Federal de Sao Paulo (UNIFESP - EPM), Sao Paulo, SP, BR.; IV INSPER Instituto de Ensino e Pesquisa, Sao Paulo, SP, BR.; V Estatikos - Assessoria e Consultoria Estatistica, Sao Paulo, SP, BR.

The high hospitalization rate of patients with coronavirus disease (COVID-19) has led to the collapse of healthcare systems in many countries. Vaccines for COVID-19 emerged in late 2020, and they have been shown to be a powerful tool to decrease hospitalization numbers in countries that were able to quickly vaccinate a majority of their populations (1).

The goal of this study was to estimate how many COVID-19-related hospitalizations were prevented in the state of São Paulo (Brazil) because of vaccines as of May 28, 2021. We used data from the SIVEP-Gripe database, which was created by the Brazilian Ministry of Health in 2019 to record deaths and hospitalized cases with severe acute respiratory illness (SARI) (2). The SIVEP-Gripe dataset is open-access and is updated weekly on the website: https://opendatasus.saude.gov.br/dataset. We used the 14-day rolling average of the daily number of patients hospitalized for SARI because of COVID-19 (SARI-COVID).

Until May 28, 2021, only individuals aged >65 years^1^ were vaccinated. Thus, we only modeled hospitalizations that were avoided in this particular group. We created a counterfactual model that used a linear regression to predict the number of SARI-COVID hospitalizations in people aged >65 years that was based on the number of hospitalizations for patients between ages 55 and 62 years (the rationale for this choice is described below). The model was estimated using only data before the first vaccination, which occurred on February 8, 2021. The counterfactual curve was then estimated by applying the fitted model to the period after February 8, 2021. Figure 1 shows the actual number of hospitalizations in patients aged >65 years, as well as the counterfactual curve with 95% prediction intervals (3) and fitted pre-vaccination model. The vertical lines indicate when the vaccination was initiated for different age groups.

Figure 1 shows that the counterfactual curve is always higher than the observed data, indicating that hospitalization levels would have been much higher if these groups had not been vaccinated. The difference between these curves became even larger in May of 2021. The reduction in hospitalizations reached its maximum of 66.1% (95% confidence interval: 65.7%, 67.5%) on May 28, 2021. The effect of vaccinations on the hospitalization rate of individuals aged >65 years is likely to improve: the Ministry of Health of Brazil has adopted a policy of using a 1-to 3-month interval between doses depending on the vaccine; thus, many individuals have only received one dose so far.

This counterfactual analysis has three main assumptions:

1. Without vaccination, the relationship between the number of hospitalizations in people aged >65 years and the younger group would have been the same after February 8, 2021.
2. Vaccination had no substantial effect on the younger group before May 28, 2021.
3. The linear model is a good approximation.

While Assumption 1 cannot be verified, we defined the age range of the younger group such that Assumptions 2 and 3 would approximately hold. Vaccination of individuals aged <62 years only started on May 6, 2021; thus, there was not enough time to observe its effect by May 28, 2021. Moreover, the blue curve in Figure 1 indicates that the linear model is a good approximation, possibly because the younger group was close in age to the group aged >65 years.

Using the areas between the curves, we observed that approximately 24,364 hospitalizations were avoided in São Paulo because of vaccination before May 28, 2021. Considering that the estimated mortality rate of hospitalized patients aged >65 years with SARI-COVID-19 is approximately 45%, approximately 10.964 deaths might have been prevented by vaccination during this period. Moreover, using estimates of the average COVID-19 hospitalization costs^2^, US $297 million may have been saved, which is enough to purchase almost 30 million additional doses of vaccine (at US $10 each).

In conclusion, we provide evidence that vaccines are an effective way to reduce mortality because of COVID-19, as well as to save substantial financial resources during the pandemic.

## Figures and Tables

**Figure 1:**
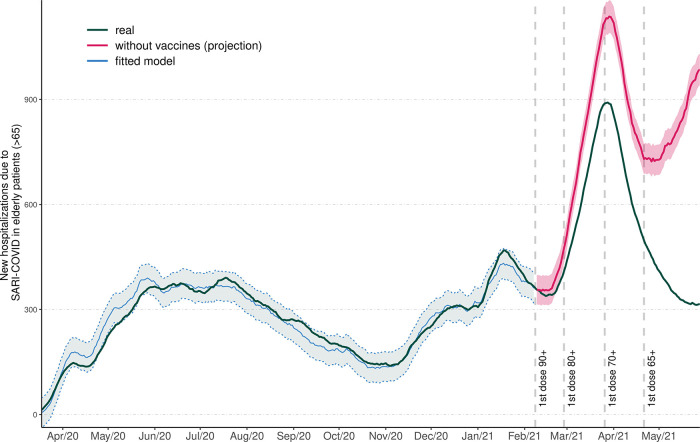
Number of hospitalizations due to SARI-COVID in patients aged >65 years (dark green), fitted pre-vaccination model (blue), and estimated counterfactual curve for the setting without vaccines (red).

## Footnotes

1 The COVID vaccination schedule of the state of São Paulo is located at https://vacinaja.sp.gov.br/

2 See https://papers.ssrn.com/sol3/papers.cfm?abstract_id=3738087
